# Quality of different obturation techniques to fill perforating internal root resorption: a micro-computed tomographic study

**DOI:** 10.1186/s12903-024-04518-w

**Published:** 2024-07-13

**Authors:** Shaimaa Mohamed Abu El Sadat, Hooi Pin Chew, Alex Fok, Mohamed Mohamed Elashiry, Ahmed M. ElShenawy, Shehabeldin Saber

**Affiliations:** 1 Department of Oral and Maxillofacial Radiology, Faculty of Dentistry, Ain Shams University, Cairo, Egypt; 2 Minnesota Dental Research Center for Biomaterials and Biomechanics, University of Minnesota, Minneapolis, USA; 3 Division of Operative Dentistry, University of Minnesota, Minneapolis, USA; 4 Department of Endodontics, The Dental College of Georgia, Augusta University, Augusta, USA; 5 Department of Endodontics, Faculty of Dentistry, Ain Shams University, Cairo, Egypt; 6 Department of Orthodontics, Faculty of Dentistry, The British University in Egypt, Cairo, Egypt; 7 Dental science Research Group. Health Research Center of Excellence, Faculty of Dentistry, The British University in Egypt, Cairo, Egypt; 8 Department of Endodontics, Faculty of Dentistry, The British University in Egypt, 81-11-11 El-Rehab, Cairo, 11841 Egypt

**Keywords:** Internal Root Resorption, Root canal perforation, Root canal Obturation, GuttaFlow-2, NeoSealer Flo, Micro-CT

## Abstract

**Background:**

This study aimed to assess the quality of various obturation techniques to fill perforation caused by internal root resorption using Micro-computed Tomography.

**Methods:**

Cone-beam computed tomography images of a maxillary central incisor tooth with perforating internal resorptive defect were used to create a 3D printed model of the affected tooth. The replicas were divided into four groups based on the obturation technique used. The techniques included Group 1: a polydimethylsiloxane-based sealer (GuttaFlow-2) with gutta-percha. Group 2: same as Group 1 except for using a pre-mixed Bioceramic-based sealer (NeoSEALER Flo). Group 3: the defect was filled entirely using the NeoSealer Flo Bioceramic-based sealer. Group 4: the samples were obturated using the warm vertical compaction technique with a resin-based sealer (ADSeal). The resin models were then scanned a micro-computed scanner to evaluate the percentage of voids in each group.

**Results:**

The results showed that NeoSEALER Flo groups had significantly the highest volume of voids while GuttaFlow-2 and warm vertical compaction groups had the lowest void volume.

**Conclusions:**

GuttaFlow-2 and warm vertical compaction techniques performed best in filling the internal resorptive defect.

## Background

Root canal anatomy may present complex irregularities in shape due to pathological processes. Internal root resorption (IRR) is an unusual condition of a tooth where the normal pulp tissue transforms into granulomatous tissues with giant cells, which resorb the dentine [1]. Internal root resorption is generally caused by infection or trauma [2]. Resorptive lacunae present a challenge when it comes to thorough disinfection and proper filling. [2, 3], which might compromise the long-term success of clinical treatment. Internal root resorption lesions might go unnoticed until the lesion reaches a considerable size, and if untreated, might result in premature loss of the affected teeth [2].

Several obturation techniques have been studied ex vivo to address obturation quality when filling IRR defects [4–6]. These studies have evaluated voids, obturation mass and the amount of gutta-percha (GP) or sealer in the defects and have shown that significant differences exist between the obturation techniques [7]. To date, there is no consensus on the best technique for obturating internal root resorption (IRR) defects. Nevertheless, warm gutta-percha (GP) has been widely utilized for this purpose. [8]. It is worth noting that GP lacks adhesion to dentin and shrinks upon cooling; thus, sealer use is mandatory [9]. In the presence of a perforated IRR defect, hydraulic calcium silicate-based materials (HCSBM) such as mineral trioxide aggregate or Biodentine are recommended owing to their recognized favorable properties such as low cytotoxicity [10], antibacterial potential [11], sealability and ability to set in the presence of blood and tissue fluids resulting in biomineralization [12]. A hybrid obturation technique is also recommended, where the root canal apical to the IRR defect is filled with GP and sealer while the IRR is filled with a HCSBM [13]. Still, it has been reported that HCSBM were associated with voids and inadequate obturation of difficult-to-access parts of the root canal system [14].

Ideally, the best material to fill IRR defects, especially in the presence of an associated perforation, should have good sealing ability and the capacity to promote healing, in addition to superior flow properties, which makes the hydraulic calcium silicate-based sealers (HCSBS) an emerging promising option for these defects [13]. Currently, limited evidence is available for the efficiency of compaction-free sealer-based obturation to fill large IRR defects.

The objective of this study was to evaluate the assess the quality of various obturation techniques to fill perforation caused by internal root resorption using Micro-computed Tomography (µCT). The null hypothesis tested was that there would be no difference in the quality of obturation in terms of voids among the different techniques.

## Methods

This study has been approved by the research ethics committee of Ain Shams University (Cairo, Egypt) under approval no. FDASU-REC PC 112,353. All methods were performed in accordance with the relevant guidelines and regulations.

### Sample size calculation

A power analysis was designed to have adequate power to apply a statistical test of the null hypothesis that there is no difference. According to the results of Torres-Carrill A.J.S. et al. [15]. and by adopting an alpha of 0.05 (5%) and a beta of 0.95 (95%), i.e., Power = 95% effect size 0.85, the predicted sample size (n) was found to be 35.

### Internal root resorption model

**Fig. 1:**
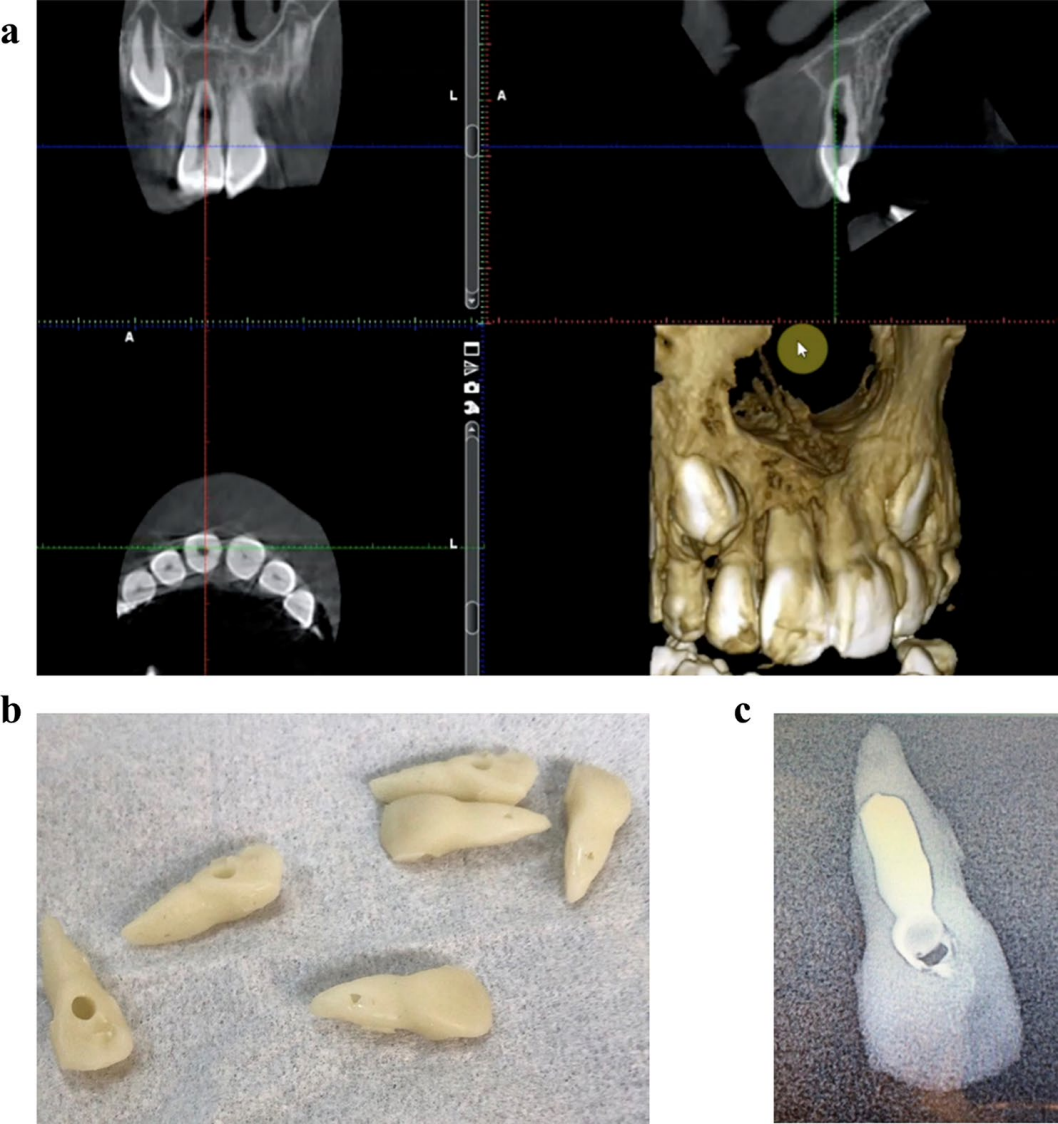
(**a)**. High-resolution CBCT scan showing the internal resorption defect. (**b).** 3D printed teeth with virtual access cavity. (**c).** Calcium hydroxide paste injected and radiographed to check the radiographic appearance of the resin material

A standardized resin model for a maxillary incisor tooth with perforating internal root resorption was obtained from high-resolution (Endo Mode) CBCT scan of a clinical case (Fig. 1a). The case was for a 9-year-old girl with a history of trauma one year ago. The patient’s chief complaint was presence of a labial sinus tract and slight tooth discoloration. The affected tooth was slightly tender to palpation and percussion, without mobility,, and responded negatively to sensibility tests. The sinus tract was radiographically traced with a gutta-percha cone size 30 and revealed that the source of infection was mid-root. Due to the suspicion of the presence of cracks in the root, a high-resolution small field-of-view CBCT was requested. The clinical diagnosis was pulp necrosis with symptomatic chronic apical periodontitis. An informed consent was obtained from the legal guardian to use the CBCT for printing of the experimental model.

The DICOM file of the CBCT scan was converted to an STL file using the Romexis software (Planmeca USA Inc., IL, USA). The STL file was fed to the DDS-PRO software to create a 3D model. A virtual access cavity was created in the crown of the 3D model of the affected tooth, no canal preparation was attempted, and finally tooth replicas were printed through additive manufacturing using a Nexdent 5100 Digital Light Processing (DLP) printer and resin Nexdent crown and bridge material shade N1 (NextDent B.V., Soesterberg, Netherlands) (Fig. 1b&c).

### Obturation

Before obturation, custom gutta-percha (GP) cones fulfilling a tug-back action were prepared by trimming 2 mm from the tip of a (size 40,4%) GP cone using a GP cutter. They were radiographically checked and selected to be the single obturating cone for groups 1,2 and 4.

Each simulated canal was irrigated with 5 ml distilled water to flush any debris. For groups 1 & 4; paper points (size 40,4%) were used to dry the samples until the last point came out dry. For groups 2 & 3; half the number of paper points were used to allow for a source of hydration of the bioceramic-based sealer.

The obturation techniques: **Group 1**: a polydimethylsiloxane-based sealer (GuttaFlow-2, Coltene/Whaledent, Langenau, Switzerland) was injected into the defect till the root canal orifice level followed by the passive insertion of the master cone, which was cut at the level of the root canal orifice using a warm pre-fitted ball burnisher. **Group 2**: same as Group 1 except for using a pre-mixed Bioceramic-based sealer (NeoSEALER Flo, Avalon Biomed, Bradenton, USA). **Group 3**: the defect was filled entirely using the NeoSealer Flo Bioceramic sealer. **Group 4**: the samples were obturated using the warm vertical compaction (WVC) technique where the GP was inserted into the canal with a resin sealer (ADSeal, Meto Biomed, Cheongju, Korea), then down packing was performed to cut the GP 5 mm shorter than the working length followed by backfilling the canal until the level of the root canal orifice.

All obturation procedures were performed under magnification by an experienced operator. After obturation, the specimens were placed in an incubator at 37 ºC and 100% humidity for 1 week to allow for complete setting. The access cavities were sealed with a light cured temporary filling.

### Micro-computed tomography analysis

#### Image acquisition

After completion of obturation procedures, the samples were scanned with micro-CT imaging system XT-H 225 (Nikon Metrology Inc., Brighton, MI, USA) for volumetric analysis of the filling materials, internal voids within the material and the external voids at the material/root canal interface. The acquisition parameters were 90–110 kV, 80–110 µA, a voxel size of 10.459 μm, and 0.5 mm Al filter.

Reconstruction parameters were defined as follows; beam hardening correction was preset to index 2, noise reduction was preset to index 1, and the volume height was adjusted to 873-934-1856 pixels. The scanning procedure took approximately 33.4 min for each scan and was performed by rotating the sample just over 180° around the vertical axis. The camera exposure time was 708 ms, and the total number of projections was 720 for each scan with 4 frames per projection. Flat field correction and geometric correction for random movement were performed in all scans.

#### Image reconstruction and quantitative assessment

The digital data were reconstructed using X-TEK CT Pro 3D (version XT 3.1.11). For calculation of the volume of the filling material and internal/external voids of the samples, the original grayscale images were processed using VG studio software **(**Version 3.4, Volume Graphics GmbH, Heidelberg, Germany**)**. The air was calibrated to define the background and the filling material was calibrated to define the material. Methodological validity was confirmed by calculating the defect volume in non-filled 3D printed samples.

**Fig. 2:**
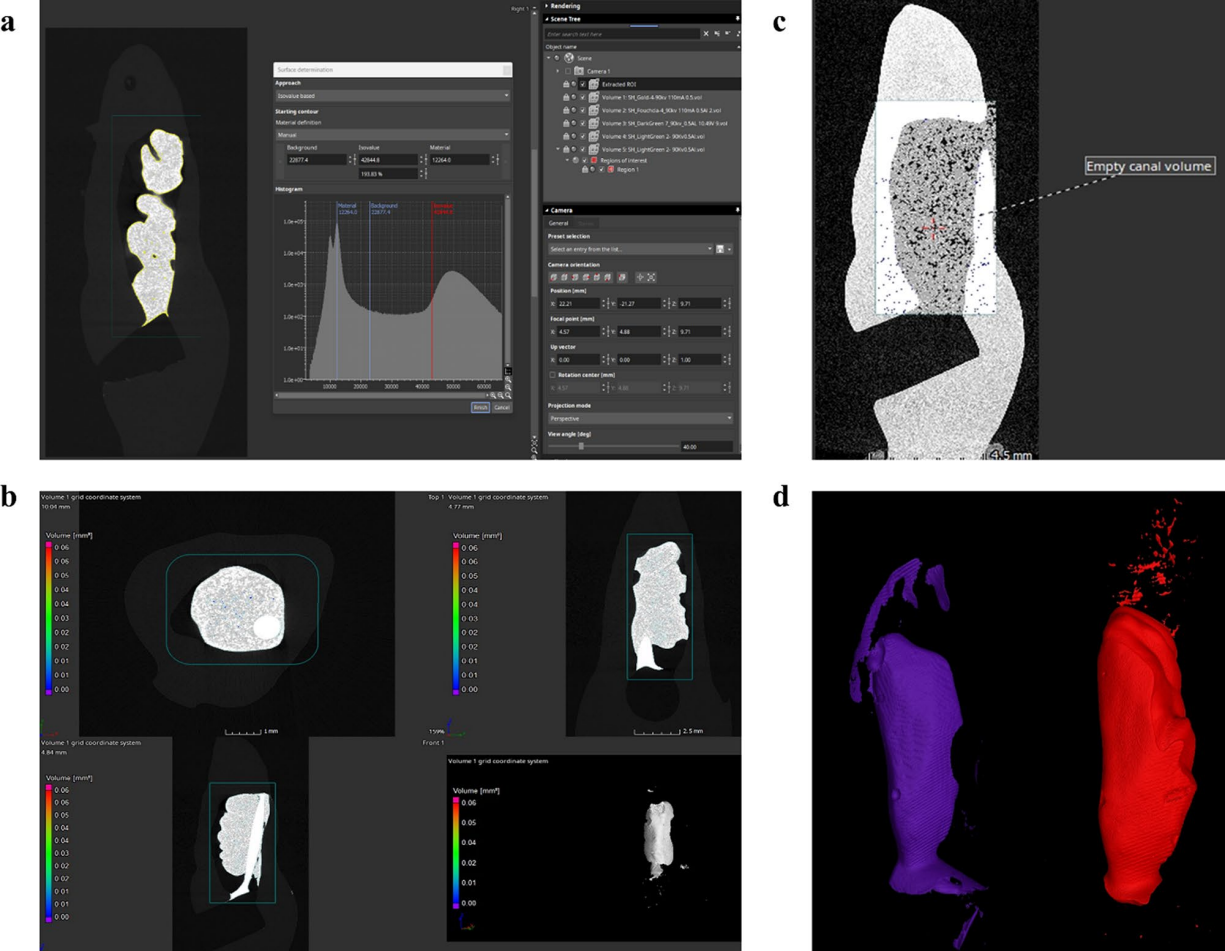
**(a)** Surface determination of the filled material with the histogram of the ROI. (**b).** MPR screen displaying the internal porosities of the bioceramic sealer with a single cone technique. (**c).** Volume calculation of empty canal. (**d).** 3D volume representation of the filling material, the purple volume is for the gutta flow and gutta percha while the red volume is for the bioceramic sealer only

A region of interest comprising the entire object was selected and then extraction of the selected area was performed. Surface determination was used to determine the volume of the material (Fig. 2a). Porosity/inclusion analysis was used to calculate the internal porosities for each material (Fig. 2b). For the control group (empty canals), the total volume was first calculated in mm^3^ (Fig. 2c). External porosities were calculated by subtracting the volume of each material from the total volume of the empty canal. The volume sum of the filling materials and the external voids was considered as the total volume, and the percentage volumes of the filling materials were calculated. Three-dimensional visualization and qualitative evaluation of the root canal obturation were performed with VG studio software (Version 3.4, Volume Graphics GmbH, Heidelberg, Germany) (Fig. 2d).

### Statistical analysis

Data were explored for normality using Kolmogorov-Smirnov and Shapiro-Wilk tests and showed a normal distribution, One-way ANOVA was performed, followed by pair-wise Tukey’s post hoc test using Graph-Pad Prism v8.1.0 (GraphPad Software, San Diego, CA, USA). Statistical significance was considered at *p* < 0.05.

## Results

### The total volume of voids

**Fig. 3:**
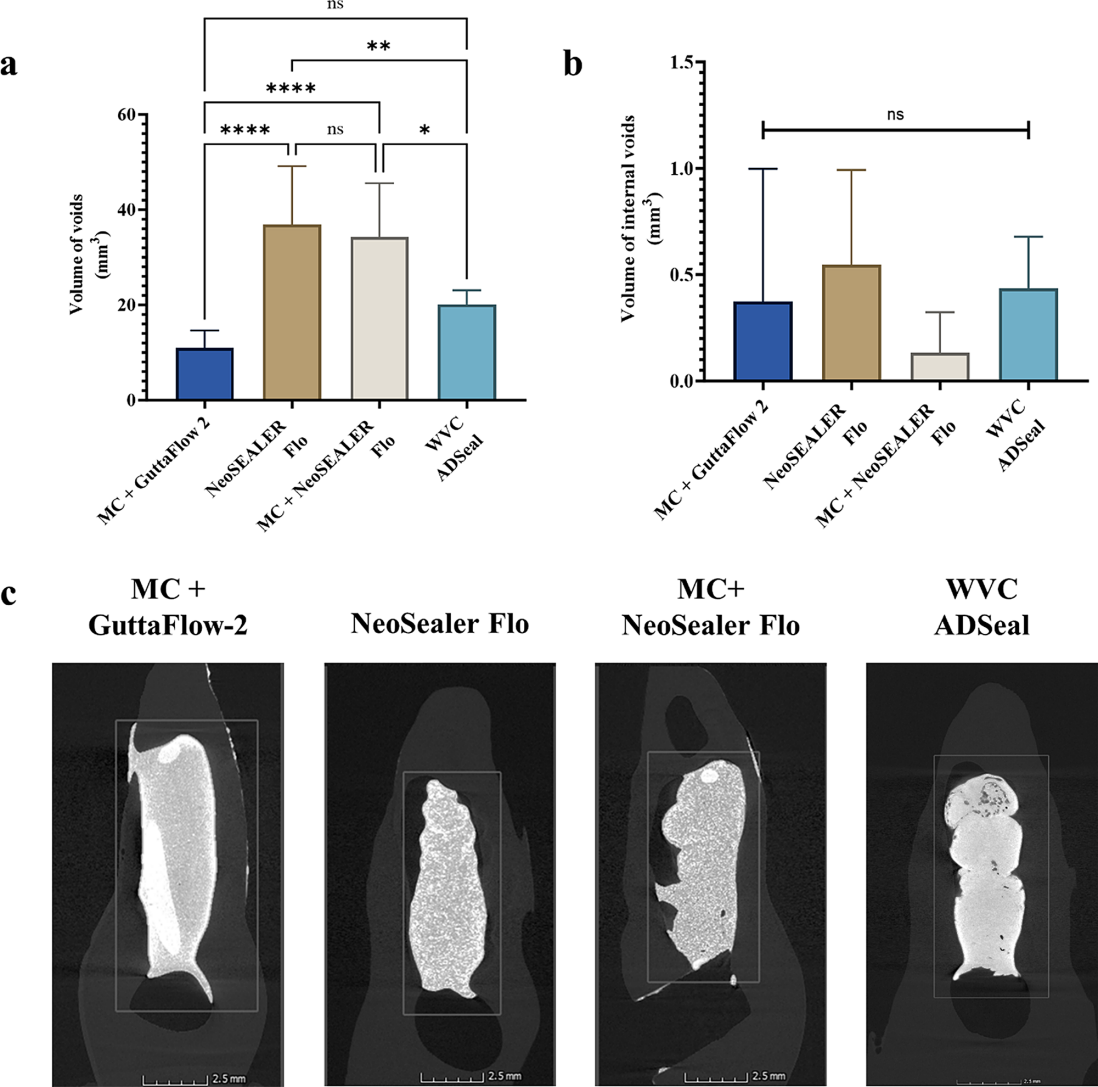
(**a).** Bar graphs showing the volume of voids between the different groups. (**b).** Bar graphs showing the volume of internal defects between the different groups. (**c).** Microcomputed tomography representative images showing the canal filling of the different groups. Data are presented as mean ± standard deviations. MC; master cone, WVC; warm vertical condensation, ns; non-significant, *p < 0.05, **p < 0.01, ****p < 0.0001

Results are shown in Table 1. The microcomputed tomography analysis of each material’s voids volume showed that the NeoSEALER Flo groups with or without master cone had significantly the highest volume of voids (Fig. 3a &c; *p* < 0.0001). GuttaFlow-2 with master cone and WVC groups had the lowest void volume (*p* < 0.0001) and were comparable to each other (*p* > 0.05).

**Table 1:** The total volume of voids after filling of the IRR defects with the different obturation techniques. Data are represented as mean ± std. Deviation

	Mean ± Std. Deviation	*p*-value
MC + GuttaFlow 2	10.96 ± 3.676^a^	*p* < 0.0001
NeoSEALER Flo	36.87 ± 12.29^b^	
MC + NeoSEALER Flo	34.30 ± 11.29^b^	
WVC ADSeal	20.06 ± 3.024^a^	

Std.; standard, MC; master cone, WVC; warm vertical condensation, different letters indicate significance

### The volume of internal voids

No significant differences were found between the groups examined regarding the volume of internal voids (Fig. 3b &c *p* = 0.2156). Data are shown in Table 2.

**Table 2:** The volume of internal voids after obturation of the IRR defects. Data are represented as mean ± std. Deviation

	Mean ± Std. Deviation	*p*-value
MC + GuttaFlow 2	0.3722 ± 0.6254	*p* = 0.2156
NeoSEALER Flo	0.5478 ± 0.4443	
MC + NeoSEALER Flo	0.1333 ± 0.1901	
WVC ADSeal	0.4363 ± 0.2423	

Std.; standard, MC; master cone, WVC; warm vertical condensation

## Discussion

Obturation of teeth with IRR defects is technically challenging, especially when it is large and perforating. To the best of our knowledge, this study is the first to prepare identical, perforated IRR models obtained from a clinical case. Previous models used acid demineralization to simulate the irregularly shaped IRR cavities [16], burs to obtain uniform cavities with well-defined borders [17] or sectioning and re-approximation of single-rooted teeth after the creation of simulated IRR defects with gates glidden drills [18]. However, anatomic standardization of samples was still difficult to achieve and validate. A recent clinical analysis of methods to evaluate obturation techniques and materials favored using of 3D-printed replicas based on models from human teeth [19].

Ideally, a flowable root canal filling material, either cold or warm, should obturate IRR defects three-dimensionally. In this study, WVC was used as the gold standard technique being reported to fill IRR defects better than other GP techniques [20]. GP has a melting interval, which is the temperature range at which it is moldable, and, hence can be mechanically compacted. [21] This thermoplasticity is the basis for the WVC technique, where GP can be compacted into canal irregularities, including IRR defects. Sealers are still required to fill gaps at the core filling material/root canal wall interface [22].

The experimental groups included GuttaFlow-2 with a single master cone and the HCSBS, NeoSealer Flo with and without a master cone. The single cone technique was selected because it is easy, time-saving and does not need special equipment. GuttaFlow-2 is a cold, silicon-based, self-cure, injectable system for canal filling that consists of gutta-percha powder with a particle size of less than 30 μm, polydimethylsiloxane, a platinum catalyst, zirconium dioxide, and micro-silver as a preservative and to add an antibacterial effect [23], while NeoSealer Flo is a single paste composed of tricalcium silicate, calcium aluminate, tantalum oxide as the radio-opacifier, and a non-aqueous liquid.

Micro-CT was used for the evaluation of the obturation quality of the root canal filling in the current study. This non-invasive imaging technique provides high-resolution images and allows three-dimensional quantitative and qualitative analysis at different greyscale levels [24]. This technology can distinguish root canal filling materials, voids, and tooth structures [25]. The presence of external and internal voids is an important indicator for assessing the marginal adaptation and density of root canal filling, respectively, and has a significant impact on treatment outcomes [26], as unfilled areas may lead to microleakage and reinfection [27].

Results of this study showed that GuttaFlow-2 and the WVC groups had the lowest void volume and were comparable to each other. The good sealing ability of the WVC technique is attributed to its hydrodynamic nature, where the master cone is down-packed and condensed. The remaining part of the root canal is back-filled with small aliquots of thermos-softened GP increments and adequately condensed, resulting in a more homogenous filling mass with good adaptation to root canal walls [28]. This agrees Karatekin et al. [29] who reported a superior efficiency for WVC in filling C-shaped canals in 3D printed resin teeth In contrast, the good sealing ability of GuttaFlow-2 is attributed to its excellent flow properties. Being thixotropic, its viscosity diminishes under pressure during placement of the master cone. Moreover, GuttaFlow-2 does not shrink but expands slightly by 0.2% upon setting. GuttaFlow-2 has been reported to fill IRR defects successfully when supplemented with a master cone [30] and has been recently shown to produce high-quality fillings in minimally-instrumented root canals [31]. Our results also showed that NeoSEALER Flo, with or without a master cone, had significantly the highest volume of voids. This finding agrees a recent integrative review reporting a superiority of WVC over single cone obturation with HCSBS regarding gaps and voids, which was attributed to lack of vertical and lateral pressure during obturation, making the filling more susceptible to acquire voids especially in the presence of challenging root canal anatomy [32]. Similar to our study, Torres-Carrillo et al. [33] evaluated the filling ability of two obturation techniques in 3D printed teeth with perforating IRR and reported a superior filling quality with an incremental technique with Bio-C putty root repair material alone, and a hybrid technique with Bio-C HCSBC/gutta-percha + Bio-C Repair. Also, Ozer et al. [34] evaluated and compared cold lateral compaction (CLC), a core carrier system (Guttacore), an injectable cold filling (GuttaFlow BioSeal) and a thermal obturation system (Elements Free) in filling IRR cavities in 3D replicas using Micro-CT and reported that GuttaFlow BioSeal and the thermal system were superior. Recently, Sharki and Ali [35] compared the percentage of obturation volume (POV) in canals with artificial IRR filled with either a single-cone technique, bulk-fill Bio-C HCSBC, and Bio-C root repair material for the coronal and middle thirds + CLC for the apical third or the thermal-based continuous wave of compaction technique. They concluded that the minimum POV was obtained with the HCSBS alone.

Debate surrounds the potential reduction of void volume associated with HCSBS over time due to its biomineralization products. Thus, further research is needed. It is worth noting that the relationship between the volume of voids within root canal fillings and treatment failure is not yet clear. Furthermore, operators should be aware that recent formulations of HCSBS have a large percentage of radio-opacifiers that can mask voids.

A limitation of this study was the interaction between the hydraulic cement and the 3D-printed teeth. The use of resin can interfere with moisture penetration necessary for hydration of CSBS. There is absence of chemical bonding, mechanical interlocking, or biomineralization formation between the plastic tooth model and bioceramic/resin sealers. However, a previous study comparing natural teeth and their printed replicas showed no differences in their percentage of voids and solubility [36]. Another limitation that can be addressed in future studies is to assess the long-term effect of different storage periods and media on the quality of the obturation techniques used to repair IRR defects, as well as their potential to reinforce endodontically treated teeth with compromised structural integrity [37].

## Conclusion

Based on the study’s limitations, none of the techniques tested provided a void-free root canal filling. However, the GuttaFlow-2 and warm vertical compaction techniques performed best in filling the internal resorptive defect with the least percentage of voids. On the other hand, the single cone technique with bioceramic-based sealers is not recommended for filling such resorptive defects.

## Data Availability

The datasets generated during and/or analyzed during the current study are available. (Attached as a related file)

## Abbreviations

IRR: Internal root resorption
GP: Gutta Percha
HCSBM: Hydraulic calcium silicate-based materials
HCSBS: Hydraulic calcium silicate-based sealers
µCT: Micro-computed Tomography
DLP: Digital Light Processing
WVC: Warm vertical compaction
CLC: Cold lateral compaction
